# GPER mediates the angiocrine actions induced by IGF1 through the HIF-1α/VEGF pathway in the breast tumor microenvironment

**DOI:** 10.1186/s13058-017-0923-5

**Published:** 2017-12-06

**Authors:** Ernestina M. De Francesco, Andrew H. Sims, Marcello Maggiolini, Federica Sotgia, Michael P. Lisanti, Robert B. Clarke

**Affiliations:** 1 Department of Pharmacy, Health and Nutritional Sciences, University of Calabria, via Savinio, 87036 Rende, Italy; 2 Breast Cancer Now Research Unit, Division of Cancer Sciences, Manchester Cancer Research Centre, University of Manchester, Wilmslow Road, Manchester, M204GJ UK; 3 Applied Bioinformatics of Cancer, University of Edinburgh Cancer Research UK Centre, Institute of Genetics and Molecular Medicine, Crewe Road South, Edinburgh, UK; 4 Translational Medicine, School of Environment and Life Sciences, Biomedical Research Centre, University of Salford, Greater Manchester, M5 4WT UK

**Keywords:** GPER, VEGF, HIF-1α, CAFs, IGF1, Breast cancer, Tumor microenvironment, Tumor angiogenesis, GPCRs, Growth factor receptors

## Abstract

**Background:**

The G protein estrogen receptor GPER/GPR30 mediates estrogen action in breast cancer cells as well as in breast cancer-associated fibroblasts (CAFs), which are key components of microenvironment driving tumor progression. GPER is a transcriptional target of hypoxia inducible factor 1 alpha (HIF-1α) and activates VEGF expression and angiogenesis in hypoxic breast tumor microenvironment. Furthermore, IGF1/IGF1R signaling, which has angiogenic effects, has been shown to activate GPER in breast cancer cells.

**Methods:**

We analyzed gene expression data from published studies representing almost 5000 breast cancer patients to investigate whether GPER and IGF1 signaling establish an angiocrine gene signature in breast cancer patients. Next, we used GPER-positive but estrogen receptor (ER)-negative primary CAF cells derived from patient breast tumours and SKBR3 breast cancer cells to investigate the role of GPER in the regulation of VEGF expression and angiogenesis triggered by IGF1. We performed gene expression and promoter studies, western blotting and immunofluorescence analysis, gene silencing strategies and endothelial tube formation assays to evaluate the involvement of the HIF-1α/GPER/VEGF signaling in the biological responses to IGF1.

**Results:**

We first determined that GPER is co-expressed with IGF1R and with the vessel marker CD34 in human breast tumors (*n* = 4972). Next, we determined that IGF1/IGF1R signaling engages the ERK1/2 and AKT transduction pathways to induce the expression of HIF-1α and its targets GPER and VEGF. We found that a functional cooperation between HIF-1α and GPER is essential for the transcriptional activation of VEGF induced by IGF1. Finally, using conditioned medium from CAFs and SKBR3 cells stimulated with IGF1, we established that HIF-1α and GPER are both required for VEGF-induced human vascular endothelial cell tube formation.

**Conclusions:**

These findings shed new light on the essential role played by GPER in IGF1/IGF1R signaling that induces breast tumor angiogenesis. Targeting the multifaceted interactions between cancer cells and tumor microenvironment involving both GPCRs and growth factor receptors has potential in future combination anticancer therapies.

**Electronic supplementary material:**

The online version of this article (doi:10.1186/s13058-017-0923-5) contains supplementary material, which is available to authorized users.

## Background

It has become increasingly recognized that the neoplastic evolution from a locally growing mass into a disseminating and aggressive metastatic disease is strongly influenced by biological events occurring in the surrounding reactive tumor stroma, which includes fibroblasts, endothelial cells and macrophages [1]. In particular, cancer-associated fibroblasts (CAFs) at the interface with cancer cells coordinate an executive biochemical program that enhances tumor progression mainly by facilitating cancer cell proliferation, migration, invasion and angiogenesis [2, 3]. In this regard, paracrine factors secreted by CAFs have been shown to trigger the formation of new blood vessels within solid tumors, thus allowing cancer cells adaptation to the local hypoxic microenvironment toward the acquisition of malignant features [4–6]. Given the evidence that tumor angiogenesis drives cancer aggressiveness and refractoriness to treatments, it is necessary to better characterise the molecular mechanisms involved in order to identify relevant targets of pharmacological intervention [7]. The vascular endothelial growth factor A (VEGF-A, also referred to as VEGF), the most notable pro-angiogenic factor, plays a key role in the generation of new blood vessel networks which provide oxygen and nutrients supply [8]. In the tumor microenvironment, the regulation of VEGF expression is mediated by the transcription factor hypoxia inducible factor-1 (HIF-1) and stimulated by low oxygen, hormones, cytokines and growth factors [8, 9]. Insulin-like growth factor 1 (IGF1) plays a pivotal role in the progression of diverse malignancies [10, 11], and has been shown to promote tumor angiogenesis by activating the HIF-1α/VEGF signaling pathway [12–16]. We have recently demonstrated that the G protein estrogen receptor (GPER) is a novel target gene of HIF-1α, involved in the regulation of VEGF in hypoxic breast tumor microenvironment [17–19]. Interestingly, GPER expression has been associated with poor clinical-pathological features in breast, endometrial and ovarian cancer patients [20–22]. GPER expression is correlated with VEGF production [23] and has been causally linked to tamoxifen resistance in breast cancer [24, 25]. We have previously demonstrated that IGF1 regulates GPER expression and function in mesothelioma and lung cancer cells [26], as well as in estrogen receptor (ER)-positive breast cancer cells [27], providing evidence that GPER may act as a further relevant contributor to the biological responses triggered by the IGF1 signaling system. In the present study, we demonstrate the strong correlation of GPER to angiocrine gene expression in breast tumours from several large cohorts totalling 4972 patients and evaluate the role of GPER in the regulation of VEGF expression and angiogenesis in GPER-positive but ER-negative primary CAFs obtained from breast tumour patients and SKBR3 breast cancer cells. We identify the molecular mechanisms through which the IGF1/IGF1R axis induces HIF-1α and GPER expression toward the stimulation of VEGF and human vascular endothelial cell tube formation.

## Methods

### Reagents

Insulin-like growth factor 1 (IGF1) was purchased from Sigma Aldrich Co Limited. Tyrphostin. AG1024 (AG) and PD98059 (PD) were purchased from Santa Cruz Biotechnology. Wortmannin (WM) was acquired from Life Technologies Ltd. SU5416 was supplied by Generon Ltd. Human VEGF was purchased from Peprotech EC: Limited. All compounds were solubilized in DMSO, except for IGF1 and VEGF, which were dissolved in water.

### Cell cultures

The SKBR3 breast cancer cells, obtained from ATCC, were maintained in DMEM (Sigma-Aldrich) with phenol red, supplemented with 10% fetal bovine serum (FBS), 1% penicillin/streptomycin and 1% Glutamax (Life Technologies). Human umbilical vein endothelial cells (HUVECs) were obtained from Sigma-Aldrich Co Limited and cultured in endothelial growth medium (EGM) (Lonza, Basel, Switzerland), supplemented with 5% FBS (Lonza). MCF7 breast and LnCAP prostate cancer cells, both obtained from American Type Culture Collection (ATCC, Manassas, VA, USA), were grown in DMEM and RPMI-1640 respectively, supplemented with 10% FBS, 1% penicillin/streptomycin and 1% Glutamax (Life Technologies). All cell lines were grown in a 37 ° C incubator with 5% CO_2_. Cells were switched to medium without serum the day before experiments.

### Isolation, cultivation and characterization of CAFs

To isolate cancer-associated fibroblasts (CAFs), signed informed consent from patients and institutional review board (IRB) approval were obtained. CAFs were isolated, cultivated and characterized as previously described [18]. Briefly, bioptic fragments from invasive mammary ductal carcinoma patients undergoing mastectomy were cut into small pieces (1 to 2 mm diameter), placed in digestion solution (400 IU collagenase, 100 IU hyaluronidase and 10% FBS, containing antibiotics and antimycotics solution) and incubated overnight at 37 °C. Cells were then separated by differential centrifugation at 90 × g for 2 minutes. The supernatant containing fibroblasts were centrifuged at 485 × g for 8 minutes, the pellet obtained was suspended in fibroblasts growth medium (Medium 199 and Ham’s F12 mixed 1:1 and supplemented with 10% FBS and 1% penicillin) and cultured at 37 °C, 5% CO_2_. In each patient, a second population of fibroblasts was isolated from a noncancerous breast tissue at least 2 cm from the outer tumor margin. CAFs and fibroblasts were then expanded into two 15-cm Petri dishes and stored as cells passaged for two to three population doublings within a total 7 to 10 days after tissue dissociation. Primary cells cultures of breast fibroblasts were characterized by immunofluorescence. In particular, cells were incubated with anti-vimentin (V9) and anti-cytokeratin 14 (LL001), obtained from Santa Cruz Biotechnology, Dallas, TX, USA. In order to assess fibroblast activation, we used anti-fibroblast activated protein α (FAPα) antibody (H-56) (Santa Cruz Biotechnology).

### Generation of stable hypoxia response element (HRE)-driven reporter SKBR3 cell line

To generate a stable HRE reporter cell line (SKBR3-HRE), which contains a luciferase gene under the transcriptional control of multiple tandemly arrayed copies of HRE sequence, the pGreenFire™ Pathway Reporter Constructs packaged in pseudotyped viral particles (Stratech Scientific Ltd) was used. Briefly, 50,000 SKBR3 cells were seeded in 24-well plate in regular growth medium for 24 h. When 50–70% confluent, medium was combined with Polybrene, before the addition of the lentiviral particles. After 72 hours, cells were subjected to selection with 2 μg/mL puromycin (Life Technologies) for 20 days. The puromycin-resistant clones were isolated and screened by measuring their basal and inducible (obtained by exposure to hypoxia, 2% O_2_) luciferase activity, as described below.

### Gene reporter assays

The 2.6 kb VEGF promoter-luciferase construct containing full-length VEGF promoter sequence (22,361 to +298 bp relative to the transcription start site) used in luciferase assays was a kind gift from Dr. P. Soumitro (Harvard Medical School, Boston, MA, USA). The GPER promoter-luciferase construct (pGPER 2.9 kb) was obtained as previously described [17]. SKBR3 (1 × 10^5^) were plated into 24-well dishes with 500 μL/well culture medium containing 10% FBS. Transfections were performed using X-treme GENE 9 DNA transfection reagent as recommended by the manufacturer (Roche Diagnostics, Basel, Switzerland, distributed by Scientific Laboratory Supplies Ltd), with a mixture containing 0.5 μg of reporter plasmid and 10 ng of pRL-TK. After 24 h, cells were treated with IGF1, as indicated. For co-transfection experiments, cells were previously transfected with control shRNA, shHIF-1α or shGPER using X-treme GENE 9 DNA transfection reagent (Roche Diagnostics, distributed by Scientific Laboratory Supplies Ltd). A mixture containing 0.5 μg of reporter plasmid and 10 ng of pRL-TK was then transfected by using X-treme GENE 9 DNA transfection reagent. After 8 hours, cells were treated for 18 hours with IGF1 in serum-free medium. Luciferase activity was measured with the Dual Luciferase Kit (Promega, Madison, WI, USA) normalized to the internal transfection control provided by Renilla luciferase activity. The normalized relative light unit values were expressed as the average fold induction of luciferase activity relative to the vehicle-treated cells, whose luciferase activity was set as 100%.

### Gene expression studies

Total RNA was extracted from cell cultures using the TRIzol commercial kit (Life Technologies) according to the manufacturer’s protocol. RNA was quantified spectrophotometrically and quality was checked by electrophoresis through agarose gels stained with ethidium bromide. Only samples that were not degraded and showed clear 18 and 28 S bands under UV light were used for RT-PCR. Total cDNA was synthesized from the RNA by reverse transcription as previously described [18]. The expression of selected genes was quantified by real-time PCR using Step One™ sequence detection system (Applied Biosystems, Foster City, CA, USA), following the manufacturer’s instructions. Gene-specific primers were designed using Primer Express version 2.0 software (Applied Biosystems) and are as follows: HIF-1α Fwd: 5’-TGCATCTCCATCTTCTACCCAAGT-3’ and Rev: 5’-CCGACTGTGAGTGCCACTGT-3’; VEGF Fwd: 5’- TGCAGATTATGCGGATCAAACC-3’ and Rev: 5’- TGCATTCACATTTGTTGTGCTGTAG-3’; GPER Fwd: 5′-CCTGGACGAGCAGTATTACGATATC-3′ and Rev 5′-TGCTGTACATGTTGATCTG-3′; 18S Fwd: 5’- GGCGTCCCCCAACTTCTTA -3’ and Rev: 5’- GGGCATCACAGACCTGTTATT -3’. Assays were performed in triplicate and the results were normalized for 18S expression and then calculated as fold induction of RNA expression.

### Western blot analysis

CAFs and SKBR3 cells were processed according to the previously described protocol [18] to obtain protein lysate that was electrophoresed through a reducing SDS/10% (w/v) polyacrylamide gel, electroblotted onto a nitrocellulose membrane and probed with primary antibodies against HIF-1α (R&D Systems, Europe Ltd), GPER (N-15), phosphorylated extracellular signal-regulated kinase (ERK) 1/2 (E-4), ERK2 (C-14), β-actin (C2), all purchased from Santa Cruz Biotechnology, pAKT (Ser 473) and AKT, which were obtained from Cell Signaling, ERα (Dako, Glostrup, Denmark), ERβ and IGF1R (Millipore). Proteins were detected by horseradish peroxidase-linked secondary antibodies (Cell Signaling) and revealed using the West Pico Chemiluminescent Substrate (Thermo Fisher Scientific, Waltham, MA, USA).

### Gene silencing experiments

Cells were plated onto 10-cm dishes and transfected using X-treme GENE 9 DNA Transfection Reagent (Roche Diagnostics, distributed by Scientific Laboratory Supplies Ltd) for 24 hours before treatments with a control shRNA, shHIF-1α, shGPER. The HIF-1α shRNA and the respective control plasmid were purchased from SABioscience Corporation. The silencing of GPER expression was obtained by the construct that we have previously described and used [28].

### Immunofluorescence assay

Fifty percent confluent cultured CAFs and SKBR3 cells grown on coverslips were serum deprived and then treated for 8 hours with IGF1 alone and in combination with AG1024, PD98059 and Wortmannin, as indicated. Where required, cells previously transfected for 24 hours with shHIF-1α or shGPER and respective negative control plasmids (as described above) and then treated for 8 hours with IGFI. Then cells were fixed in 4% paraformaldehyde, permeabilized with 0.2% Triton X-100, washed three times with PBS and incubated overnight with a mouse primary antibody against VEGF (C-1) (Santa Cruz Biotechnology). After incubation, the slides were extensively washed with PBS, probed with donkey anti-mouse IgG-FITC (1:300; purchased from Alexa Fluor, Life Technologies) and 4′,6-diamidino-2-phenylindole dihydrochloride (DAPI) (Sigma-Aldrich Co Limited) and then imaged on a fluorescence microscope.

### Conditioned medium

CAFs and SKBR3 cells were cultured in regular growth medium, then cells were washed twice with PBS and transfected for 24 hours in serum-free medium with shHIF-1α, shGPER or control shRNA using X-treme GENE 9 DNA Transfection Reagent (Roche Diagnostics, distributed by Scientific Laboratory Supplies Ltd). Cells were treated for 8 hours with IGF1, culture medium was then replaced for additional 12 hours with medium without serum. Thereafter, the supernatants were collected, centrifuged at 3500 rpm for 5 minutes to remove cell debris and used as conditioned medium in HUVECs.

### Tube formation assay

The day before the experiment, confluent HUVECs were starved overnight at 37 °C in serum-free medium. Growth factor-reduced Matrigel® (R&D Systems Europe Ltd) was thawed overnight at 4 °C on ice, plated on the bottom of pre-chilled 96-well plates and left at 37 °C for 1 hour for gelification. Starved HUVECs were collected by enzymatic detachment (0.25% trypsin-EDTA solution, Life Technologies), counted and resuspended in conditioned medium from CAFs or SKBR3 cells. Then, 10,000 cells/well were seeded on Matrigel and incubated at 37 °C. Tube formation was observed starting from 4 hours after cell seeding and quantified by using the software NIH ImageJ (National Institutes of Health (NIH), Bethesda, MD, USA).

### Gene expression signature in breast cancer patients

Gene expression levels of GPER and IGF1 signaling-related genes were retrieved from the Molecular Taxonomy of Breast Cancer International Consortium (METABRIC) dataset [29] and from integrating 17 Affymetrix gene expression datasets as previously described [30]. Gene expression data from both Affymetrix and METABRIC studies were representative of both ER-positive (+) and ER-negative (-) breast tumours. Similarly, Affymetrix gene expression data from three breast cancer cell line panel studies were integrated as described previously [30].

### Statistical analysis

Statistical analysis was performed using *t* tests and Spearman correlations. *p* < 0.05 was considered statistically significant.

## Results

### GPER and IGF1R define an angiocrine signature in breast tumor patients

**Fig. 1:**
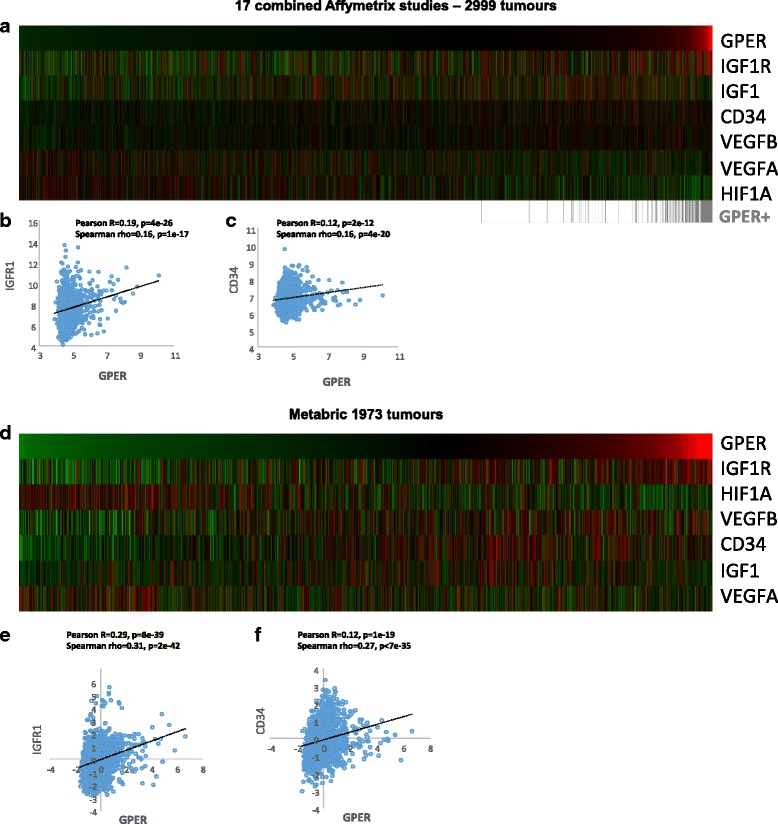
GPER correlates with IGF1R and CD34 expression in breast tumor samples. Data showing angiocrine-related genes across the 17 study integrated Affymetrix (**a**-**c**) and METABRIC (**d**-**f**) datasets of 2999 and 1973 breast cancer patients, respectively. In the heatmaps, ranked *from left to right* GPER expression correlates with IGF1R and the angiogenic marker CD34. Colors are log2 mean-centered values; *red* indicates high, and *green* indicates low. Tumours in which GPER is called ‘present’ (GPER+) using the MAS5 detection calls are indicated in *grey. GPER* G-protein estrogen receptor, *HIF-1* hypoxia inducible factor-1, *IGF1* insulin-like growth factor 1, *VEGF* vascular endothelial growth factor

In order to evaluate whether GPER is involved in the angiogenic actions elicited by the IGF1 system in breast cancer, we analyzed gene expression from 17 published Affymetrix microarray tumor gene expression datasets of 2999 breast cancer patients [30] and METABRIC [29], a second independent dataset of 1973 breast tumor samples analysed for gene expression using Illumina BeadChips. Our analysis considered a panel of genes implicated in angiogenesis and correlated them with the expression of GPER. In both datasets, a strong, positive correlation in expression was found between GPER and IGF1R, and between GPER and the microvessel density marker CD34 [31] (Fig. 1), indicating that IGF1R and GPER may be involved in angiocrine regulation of the breast tumor microenvironment. In contrast, IGF1, VEGFA and VEGFB were not significantly correlated with GPER expression, and a negative correlation was detected between the expression of GPER and HIF-1α (Fig. 1). These large, independent patient datasets, comprising 4972 breast tumors, suggested to us that GPER, IGF1/IGF1R signaling and blood vessel density (CD34) are correlated, and that GPER and IGF signaling may be linked to changes in the breast tumor microenvironment.

### IGF1 induces HIF-1α, GPER and VEGF expression to stimulate angiocrine signaling by the breast tumour microenvironment

Based on the above correlations, we investigated the cross-talk between GPER and IGF1/IGF1R system in mediating tumor angiogenesis in vitro. To model the breast tumor microenvironment, we used cancer-associated fibroblasts (CAFs) isolated from breast tumor patients (see Methods), and in addition, the SKBR3 breast cancer cell line, which is classed as luminal by gene expression analysis (Additional file 1: Figure S1A). This analysis of unsupervised clustering of three integrated breast cell line datasets showed a strong correlation between IGF1R and GPER expression in the luminal cell lines (Additional file 1: Figure S1B).

**Fig. 2:**
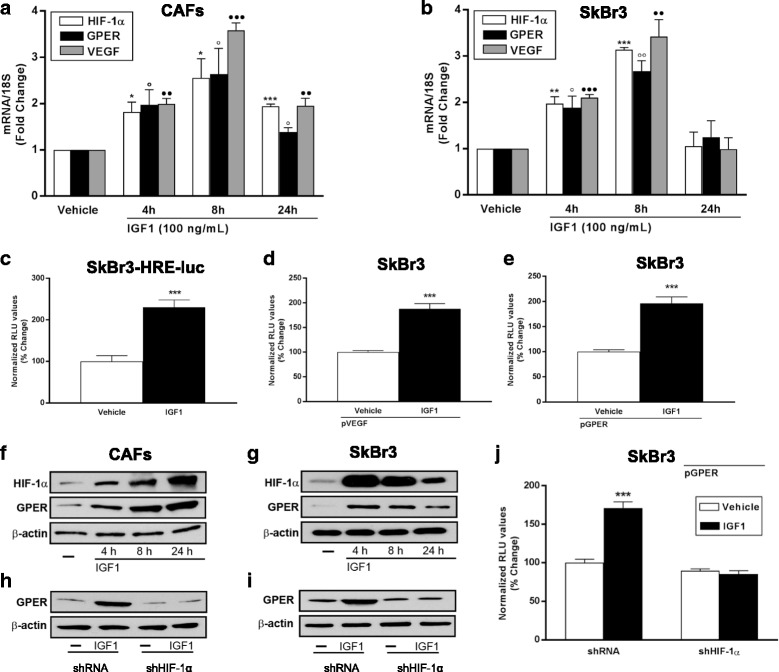
IGF1 induces the expression of HIF-1α, GPER and VEGF*.* mRNA expression of HIF-1α, GPER and VEGF in CAFs (**a**) and SKBR3 (**b**) cells treated for 8 hours with 100 ng/mL, as evaluated by real-time PCR. Values are normalized to the 18S expression and shown as fold changes of mRNA expression induced by IGF1 compared to cells treated with vehicle. Data are mean ± SEM of three independent experiments performed in triplicate. **c** Evaluation of luciferase activity in SKBR3 cells infected with a HRE reporter construct (SKBR3-HRE-luc) and treated for 18 hours with IGF1 (100 ng/mL). The luciferase activities were normalized to the protein content, evaluated in parallel plate by SRB (sulforhodamine B) assay. The transactivation of a VEGF (pVEGF) (**d**) and a GPER (pGPER) (**e**) promoter construct is observed in SKBR3 cells treated with 100 ng/mL IGF1 for 18 hours. Luciferase activity was normalized to the internal transfection control. Results are expressed as the % change of normalized RLU values relative to vehicle-treated cells. Data shown are the mean ± SEM of two independent experiments performed in triplicate. Representative immunoblots showing the increase of HIF-1α and GPER protein expression in CAFs (**f**) and SKBR3 cells (**g**) treated with 100 ng/mL IGF1 for 8 hours. The upregulation of GPER protein expression observed treating CAFs (**h**) and SKBR3 cells (**i**) for 8 hours with 100 ng/mL IGF1 is abrogated by silencing HIF-1α. β-actin serves as loading control. **j** The transactivation of a GPER promoter construct (pGPER) detected in SKBR3 cells treated with 100 ng/mL IGF1 for 18 hours is abrogated by HIF-1α silencing. (*), (○), *p* < 0.05; (**), (○○), (●●) *p* < 0.01; (***), (●●●) *p* < 0.001. *CAFs* cancer-associated fibroblasts, *GPER* G-protein estrogen receptor, *HIF-1* hypoxia inducible factor-1, *IGF1* insulin-like growth factor 1, *VEGF* vascular endothelial growth factor

In addition, both CAFs and SKBR3 cells express GPER and IGF1R, but not ERs (Additional file 2: Figure S2A), thus allowing us to investigate the interactions between GPER and the IGF1/IGF1R axis, without any contribution and/or interference from ER signaling. In order to investigate the angiocrine actions of IGF1 in breast tumour, first of all we asked whether IGF1 induces the expression of VEGF and its transcriptional regulator HIF-1α in our experimental models. IGF1 upregulated the mRNA expression of HIF-1α and VEGF (Fig. 2a-b), as determined performing RT-PCR experiments in both CAFs and SKBR3 cells. Further corroborating these findings, IGF1 increased the luciferase activity of SKBR3 cells engineered to stably express a hypoxia response element (HRE)-driven reporter construct (SKBR3-HRE-luc) (Fig. 2c); in addition, the transactivation of a VEGF promoter construct (pVEGF) was observed in SKBR3 cells stimulated with IGF1 (Fig. 2d). Next, we assessed whether GPER is involved in the aforementioned stimulatory responses induced by IGF1. In both CAFs and SKBR3 cells, IGF1 induced the upregulation of GPER mRNA levels (Fig. 2a-b) and transactivated a GPER promoter construct (pGPER) (Fig. 2e). At the protein level, IGF1 induced HIF-1α and GPER expression in a time-dependent manner, as determined by western blotting experiments performed in both CAFs and SKBR3 cells (Fig. 2f-g and Additional file 2: Figure S2b-c). Furthermore, the upregulation of GPER protein expression (Fig. 2h-i and Additional file 2: Figure S2 D-E) and the transactivation of a GPER promoter construct (Fig. 2j) induced by IGF1 were prevented in the presence of HIF-1α knockdown (Additional file 3: Figure S3 A-C), indicating that GPER is induced by IGF1 via HIF-1α transcriptional activity.

Altogether, these findings suggest that HIF-1α and its transcriptional targets GPER and VEGF are triggered by IGF1 both in breast tumor microenvironmentally derived CAFs and in luminal breast cancer cells.

### Molecular mechanisms of the angiocrine activity of IGF1

**Fig. 3:**
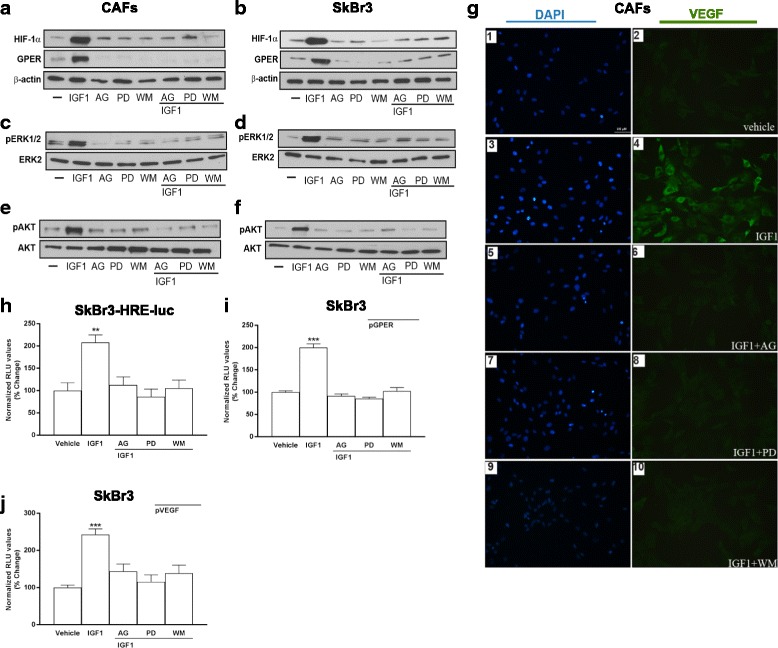
ERK1/2 and AKT signaling pathways are involved in the upregulation of VEGF expression induced by IGF1. The upregulation of HIF-1α and GPER protein expression observed treating CAFs (**a**) and SKBR3 cells (**b**) with 100 ng/mL IGF1 for 8 hours is abolished in the presence of 10 μM IGF1R inhibitor AG1024 (AG), 10 μM MEK inhibitor PD98059 (PD) and 100 nM PI3K inhibitor Wortmannin (WM). The activation of ERK1/2 and AKT (Ser 473) is prevented in CAFs (**c**, **e**) and SKBR3 cells (**d**, **f**) treated for 60 minutes with 100 ng/mL IGF1, alone and in combination with AG (10 μM), PD (10 μM) and WM (100 nM). ERK2, AKT and β-actin serve as loading control, as indicated. **g** VEGF protein expression in CAFs treated with 100 ng/mL IGF1 for 8 hours, alone and in combination with AG (10 μM), PD (10 μM) and WM (100 nM), as evidenced by immunfluoerscence experiment. VEGF accumulation is shown by the *green signal*, nuclei are stained by DAPI (*blue signal*), bar scale 100 μM. Results shown are representative of two independent experiments. Evaluation of luciferase activity in SKBR3 cells infected with a HRE reporter construct (SKBR3-HRE-luc) (**h**), and in SKBR3 cells transiently transfected with a GPER (pGPER) (**i**) or a VEGF (pVEGF) promoter construct (**j**) and treated with 100 ng/mL IGF1 for 18 h in the presence of AG, PD and WM. Luciferase activity was normalized to the internal transfection control. Results are expressed as the % change of normalized RLU values relative to vehicle-treated cells. Each data point represents the mean ± SEM of two independent experiments performed in triplicate. (**) *p* < 0.01; (***) *p* < 0.001. *CAFs* cancer-associated fibroblasts, *GPER* G-protein estrogen receptor, *HIF-1* hypoxia inducible factor-1, *IGF1* insulin-like growth factor 1

Previously, ERK1/2 and AKT signaling cascades have emerged as pivotal transduction mediators involved in IGF1-induced and HIF-1-dependent responses in cancer cells [32, 33]. On the basis of these observations, we determined that the upregulation of HIF-1α and GPER protein expression induced by IGF1 was repressed in the presence of the IGF1R tyrosine kinase inhibitor AG1024 (AG), the MEK inhibitor PD98059 (PD) as well as the PI3K inhibitor Wortmannin (WM) (Fig. 3a-b and Additional file 4: Figure S4 A,D). Accordingly, the luciferase activity of SKBR3 cells engineered to stably express a hypoxia response element (HRE)-driven reporter construct (SKBR3-HRE-luc) and the transactivation of a GPER promoter construct observed upon treatment with IGF1 were prevented by AG, PD and WM (Fig. 3h-i). Of note, these three pharmacological inhibitors were also able to block the increase of VEGF protein expression together with the transactivation of a VEGF promoter construct induced by IGF1, as demonstrated by immunofluorescence experiments and luciferase assays respectively (Fig. 3g, j and Additional file 5: Figure S5). Corroborating these findings, in CAFs and SKBR3 cells stimulated with IGF1 the activation of ERK1/2 and AKT was prevented by AG, PD and WM (Fig. 3c-f and Additional file 4: Figure S4 B,C,E,F). Taken together these findings suggest that IGF1/IGF1R axis engages ERK1/2 and AKT signaling to trigger the activation of HIF-1α/GPER/VEGF transduction pathway in breast tumor microenvironment.

**Fig. 4:**
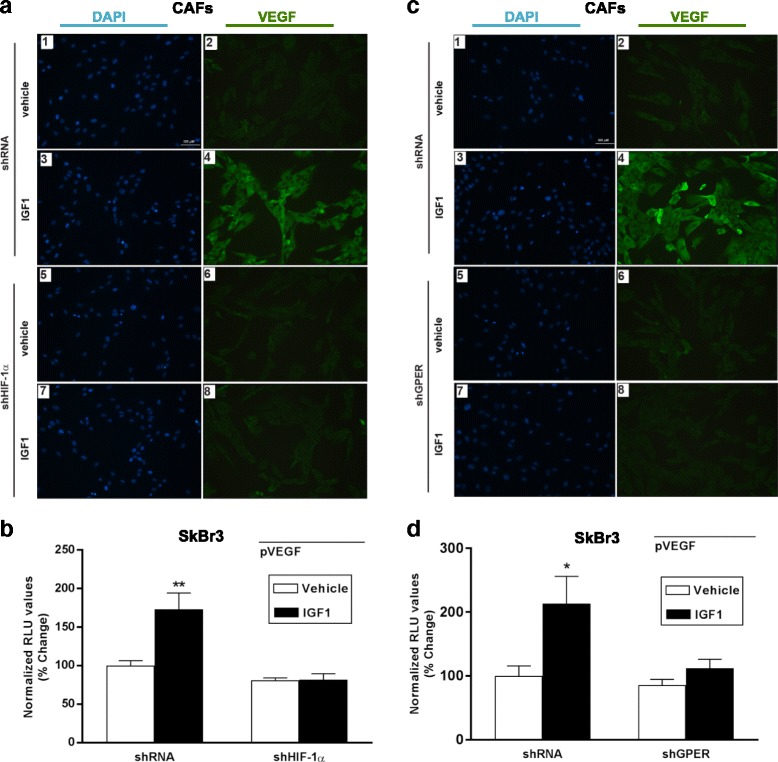
HIF-1α and GPER are involved in VEGF protein increase induced by IGF1. **a** Evaluation of VEGF protein expression by immunofluorescence experiment in CAFs transfected for 24 hours with control shRNA (*panels 1–4*) or shHIF-1α (*panels 5–8*) and treated with 100 ng/mL IGF1 for 8 hours, as indicated. **b** The transactivation of a VEGF (pVEGF) promoter plasmid observed in SKBR3 cells treated with 100 ng/mL IGF1 for 18 hours is abrogated silencing the expression of HIF-1α. **c** Evaluation of VEGF protein expression by immunofluorescence experiment in CAFs transfected for 24 hours with control shRNA (*panels 1–4*) or shGPER (*panels 5–8*) and treated with 100 ng/mL IGF1 for 8 hours, as indicated. In immunofluorescence experiments, VEGF accumulation is evidenced by the *green* signal, nuclei are stained by DAPI (*blue signal*), bar scale 100 μM. Images shown are representative of two independent experiments. **d** The transactivation of a VEGF (pVEGF) promoter plasmid observed in SKBR3 cells treated with 100 ng/mL IGF1 for 18 hours is abrogated silencing the expression of GPER. In luciferase assays, luciferase activity was normalized to the internal transfection control. Results are expressed as the % change of normalized RLU values relative to vehicle-treated cells. Each data point represents the mean ± SEM of two independent experiments performed in triplicate. (*) *p* < 0.05, *p* < 0.01 (**). *CAFs* cancer-associated fibroblasts, *GPER* G-protein estrogen receptor, *HIF-1* hypoxia inducible factor-1, *IGF1* insulin-like growth factor 1, *VEGF* vascular endothelial growth factor

Our previous investigations have evidenced the functional cooperation between HIF-1α and GPER in the regulation of VEGF expression and angiogenesis in hypoxic breast tumor microenvironment [18]. Thus, we sought to address whether both HIF-1α and GPER are required for the regulation of VEGF expression induced by IGF1. In CAFs and SKBR3 cells, IGF1 failed to increase VEGF protein expression (Fig. 4a and Additional file 6: Figure S6 A-B) and activate a VEGF promoter construct (Fig. 4b) in the presence of HIF-1α knockdown (Additional file 3: Figure S3 D-E). Interestingly, gene silencing experiments demonstrated that also GPER is required for the upregulation of VEGF protein expression (Fig. 4c, Additional file 3: Figure S3F and Additional file 6: Figure S6 C-D) and the transactivation of a VEGF promoter construct induced by IGF1 (Fig. 4d and Additional file 3: Figure S3 G). Collectively, these data provide evidence on the molecular mechanisms activated by IGF1 through GPER toward the regulation of VEGF expression in breast cancer cells.

### IGF1 regulates HIF-1α/GPER signaling to trigger VEGF-mediated endothelial tube formation

**Fig. 5:**
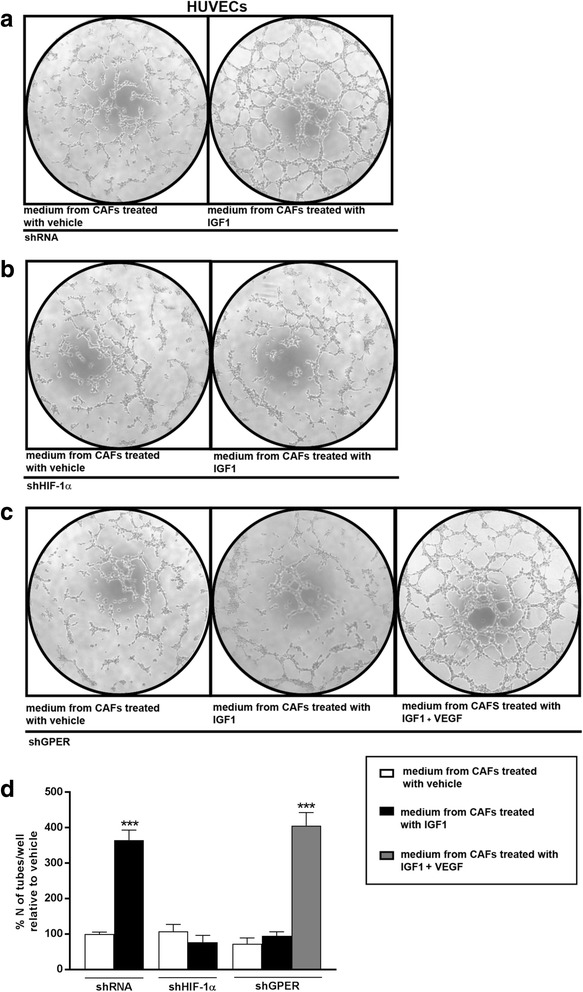
HIF-1α and GPER are involved in the formation of endothelial tubes mediated by VEGF. Tube formation in HUVECs cultured in medium collected from CAFs treated with vehicle or 100 ng/ml IGF1 for 18 hours; CAFs were transfected for 24 hours with control shRNA (**a**), shHIF-1α (**b**) or shGPER (**c**) before adding treatments. **c** 10 ng/mL VEGF rescues tube formation in HUVECs cultured in conditioned medium from GPER-silenced CAFs, which were treated with 100 ng/mL IGF1 for 18 hours. **d** Quantification of the number of tubes, observed in HUVECs, as indicated. Data are representative of three independent experiments performed in triplicate. (***) *p* < 0.001. *CAFs* cancer-associated fibroblasts, *GPER* G-protein estrogen receptor, *HIF-1* hypoxia inducible factor-1, *HUVECs* human umbilical vein endothelial cells, *IGF1* insulin-like growth factor 1, *VEGF* vascular endothelial growth factor

**Fig. 6:**
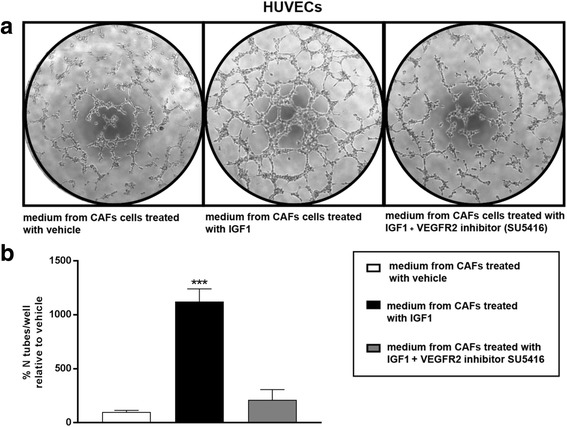
VEGF triggers endothelial tube formation via VEGFR2. **a** Endothelial tube formation is abrogated in HUVECs cultured for 4 hours in medium collected from CAFs treated with 100 ng/mL IGF1 for 18 hours, in the presence of the VEGFR2 inhibitor SU5416 (1 μM). **b** Quantification of the number of tubes observed in HUVECs, as indicated. Data are representative of three independent experiments performed in triplicate. (***) *p* < 0.001. *CAFs* cancer-associated fibroblasts, *HUVECs* human umbilical vein endothelial cells, *IGF1* insulin-like growth factor 1, *VEGF* vascular endothelial growth factor

On the basis of the findings above that indicate that a functional interplay between HIF-1α and GPER regulates the expression of the key angiogenic mediator VEGF, we next evaluated the contribution of HIF-1α/GPER signaling to the stimulatory response induced by IGF1 in breast tumor microenvironment. Conditioned medium obtained from CAFs and SKBR3 cells was collected and used in HUVEC tube formation assay in Matrigel. As shown in Fig. 5, a complex and ramified network of tubules was detected in HUVECs cultured in conditioned medium from CAFs treated with IGF1 (panel A), while this effect was no longer evident in the presence of HIF-1α and GPER silencing (panels B-C and Additional file 3: Figure S3 H-I). However, a complete rescue of tubulogenesis was observed when VEGF was added to the conditioned medium collected from GPER-silenced and IGF1-treated CAFs (Fig. 5c). These data were quantified in Fig. 5d and Additional file 7: Figure S7 A-B. Analogous results were obtained performing tube formation assay using HUVECs cultured with conditioned medium collected from SKBR3 cells (Additional file 8: Figure S8). In addition, the efficiency of endothelial tube formation was significantly reduced when the VEGFR2 inhibitor SU5416 was added to the conditioned medium obtained from CAFs (Fig. 6a-b and Additional file 7: Figure S7 C-D) and SKBR3 cells (Additional file 9: Figure S9) treated with IGF1. Altogether, these results establish that HIF-1α/GPER signaling is required downstream of IGF1 for VEGF-mediated angiogenesis in the breast tumor microenvironment.

## Discussion

In the current study, we investigated the role of GPER in the regulation of VEGF expression and tumor angiogenesis induced by IGF1. We establish in primary patient-derived breast CAFs, and in luminal breast cancer cells that IGF1 triggers the expression of VEGF and its transcriptional regulators HIF-1α and GPER, through the ERK1/2 and AKT transduction pathways. In addition, we provide evidence that both HIF-1α and GPER contribute to the regulation of VEGF stimulated by IGF1. In biological assays, we showed that IGF1 requires both HIF-1α and GPER to induce VEGF-mediated endothelial tube formation, as evidenced by HUVECs cultured with conditioned medium obtained from CAFs and breast cancer cells treated with IGF1. Interestingly, in human breast tumor samples, GPER expression correlates with IGF1R and with the vessel marker CD34, corroborating the engagement of GPER and IGF1 system in breast tumor angiogenesis in breast tumor microenvironment.

These findings are in accordance with previous evidence obtained both in vitro and in vivo indicating that IGF1 triggers VEGF-mediated neovascularization in breast, endometrial, head and neck, lung, colon cancer and sarcoma cells [13–16, 32, 34].

In this regard, the stimulatory action elicited by IGF1 has been particularly described for estrogen receptor (ER)-positive breast tumors, where a cross-talk between IGF1R and ER transduction pathways has been shown to regulate critical biological responses like cancer cell proliferation and migration [35–38]. In the present study we used as model systems patient-tumor derived CAFs and SKBR3 breast cancer cells, which allowed us to characterize the IGF1/IGF1R action in ER-negative breast cancer cell setting. It should be mentioned that a 36 kDa splice variant of ERα named ERα36 has been shown to be expressed in SKBR3 cells [39], however our data demonstrate that GPER knockdown in SKBR3 cells abrogates the stimulatory responses to IGF1, thus indicating that GPER is required for the stimulatory actions induced by IGF1.

We found that the IGF1/IGF1R transduction pathway through the involvement of ERK1/2 and AKT signaling cascades triggers an angiogenic gene signature characterized by the increase of VEGF as well as its transcriptional regulator HIF-1α. These data are well supported by previous evidence showing that the angiocrine action exerted by IGF1 through IGF1R occurs through the activation of HIF-1α/VEGF axis and the involvement of MAPK and PI3K/AKT activity [32]. Importantly, stimuli other than hypoxia like cytokines, chemokines, hormones and growth factors have been reported to trigger HIF-1-dependent responses through non-canonical mechanisms [40]. In this regard, IGF1 has been shown to induce HIF-1α protein synthesis at the translational level, without affecting HIF-1 gene transcription or protein stability in colon cancer cells [32] and an increase in HIF-1α mRNA levels has been demonstrated after stimulation with IGF1 in several cells models [41–43]. Our results support these studies, showing that IGF1/IGF1R axis triggers HIF-1 expression at the mRNA level leading to the transcription of target genes.

Herein, we also show that IGF1 activates the HIF-1α-dependent expression of GPER, required for the regulation of VEGF in CAFs and breast cancer cells. GPER, which is considered as a negative prognostic marker in breast cancer [21], has been shown previously to be upregulated and/or activated by a plethora of stimuli classically involved in HIF-1 pathway, including hypoxia, EGF, IGF1, insulin and the metals copper and zinc [2–27, 44–48]. In addition, in breast cancer cells and CAFs as well as in a mouse model of breast cancer, estrogenic GPER signaling has been shown to trigger HIF-1α/VEGF pathway leading to angiogenesis and tumor growth [48, 49]. In this context, data shown herein, indicating that GPER triggers VEGF expression and function, are strengthened by previous investigations demonstrating that GPER overexpression is associated with high VEGF production rates in primary cell cultures derived from endometrial cancer tissues [23]. Furthermore, our data regarding the integrated bioinformatic analysis of gene expression studies from human breast tumor samples, evidence that GPER and the IGF1/IGFIR system may be regarded as a peculiar angiocrine signature, for their co-expression pattern with the microvessel-density marker CD34 [31]. It should be mentioned that in our gene data, obtained from the integrated bioinformatic analysis of human breast tumor samples, IGF1 and VEGF are not positively correlated with the expression of GPER (Fig. 1). In addition, in contrast with our in vitro findings, we detected a negative correlation between HIF-1α and GPER gene expression in breast tumor samples. Such discrepancies could be due to intrinsic limits of the gene expression technique used, as well as to the relative contribution of the diverse cellular components present in the breast tumor samples, including macrophages, immune cells and adipocytes. In addition, HIF-1α expression has been shown to be tightly regulated by local oxygen levels, which may be fluctuating in the tumor mass due to cycling hypoxia; moreover, the fluctuation of HIF-1α expression in response to oxygen or other stimuli are peculiarly and primarily regulated at the protein level, as HIF-1α protein stabilization occurs before mRNA transcription [50, 51]. It should also be considered that the mechanisms and factors involved in HIF-1α regulation are cell-type specific [52], while the data shown in our bioinformatics analysis are representative of the whole tumor tissue.

Nonetheless, our findings indicate that GPER and the IGF1/IGF1R axis are co-expressed in human breast tumors, suggesting that targeting IGF1R/GPER cross-talk might be useful in halting the angiogenesis process, particularly in a subset of breast cancer patients devoid of the classic ER. On the basis of literature data and our present results, GPER may be included among the transduction mediators involved in neovascularization triggered by HIF-1α/VEGF signaling axis in tumor microenvironment. The current study corroborate the role of GPER in the complex process of tumor angiogenesis induced by IGF1, supporting the idea that the aberrant cross-talk between receptor tyrosine kinases (RTKs)- and G-protein coupled receptors (GPCR)-mediated signaling may converge on relevant biological responses driven by HIF-1 toward VEGF-dependent new blood vessel formation.

## Conclusions

Overall, our study provides novel evidence regarding the angiocrine action elicited by IGF1 in breast cancer through the activation of HIF-1α/VEGF signaling pathway, emphasizing the important role played by GPER in the modulation of this relevant transduction axis. A better understanding of the effects induced by IGF1 through GPER at the crossroad between cancer, endothelial and stromal cells, may pave the way for the therapeutic manipulation of the multiple signaling networks that control tumor angiogenesis. Although further validation of these results is required in additional tumor models, our study suggests that reprogramming the cross-talk between RTKs and GPCRs in the vascular niche may represent a promising novel approach to control aberrant angiogenesis in cancer patients.

## Additional files

Additional file 1: Figure S1. GPER and IGF1R co-expression in luminal breast cancer cell lines. (PDF 167 kb)

Additional file 2: Figure S2. Estrogen receptor expression and densitometric analysis of western blotting. (PDF 1130 kb)

Additional file 3: Figure S3. Efficacy of HIF-1α and GPER silencing. (PDF 3483 kb)

Additional file 4: Figure S4. ERK1/2 and AKT activation in SKBR3 cells and CAFs. (PDF 155 kb)

Additional file 5: Figure S5. IGF1 induces VEGF protein expression in CAFs. (PDF 1335 kb)

Additional file 6: Figure S6. HIF-1α and GPER silencing abrogates IGF1-induced VEGF expression in SKBR3 cells. (PDF 2451 kb)

Additional file 7: Figure S7. Quantification of tube formation. (PDF 123 kb)

Additional file 8: Figure S8. IGF1 triggers endothelial tube formation. (PDF 1076 kb)

Additional file 9: Figure S9. VEGFR2 inhibition prevents endothelial tube formation. (PDF 457 kb)

## Abbreviations

CAFs: Cancer-associated fibroblasts
EGF: Epidermal growth factor
ER: Estrogen receptor
ERK: Extracellular signal-regulated kinase
GPCRs: G-protein coupled receptors
GPER: G-protein estrogen receptor
HIF-1: Hypoxia inducible factor-1
HRE: hypoxia response element
HUVECs: human umbilical vein endothelial cells
IGF1: Insulin-like growth factor 1
RTKs: Receptor tyrosine kinases
VEGF: Vascular endothelial growth factor
