# Supplementary material for: GPER mediates the angiocrine actions induced by IGF1 through the HIF-1α/VEGF pathway in the breast tumor microenvironment
Source: Breast Cancer Res. 2017 Dec 6;19:129. doi: 10.1186/s13058-017-0923-5 (PMC5719673; doi:10.1186/s13058-017-0923-5)
Supplement: Supplementary file 1 — GPER and IGF1R co-expression in luminal breast cancer cell lines. (PDF 167 kb) [file 13058_2017_923_MOESM1_ESM.pdf]

Unsupervised clustering (500 most variable genes)
of 3 integrated breast cell line datasets.

A

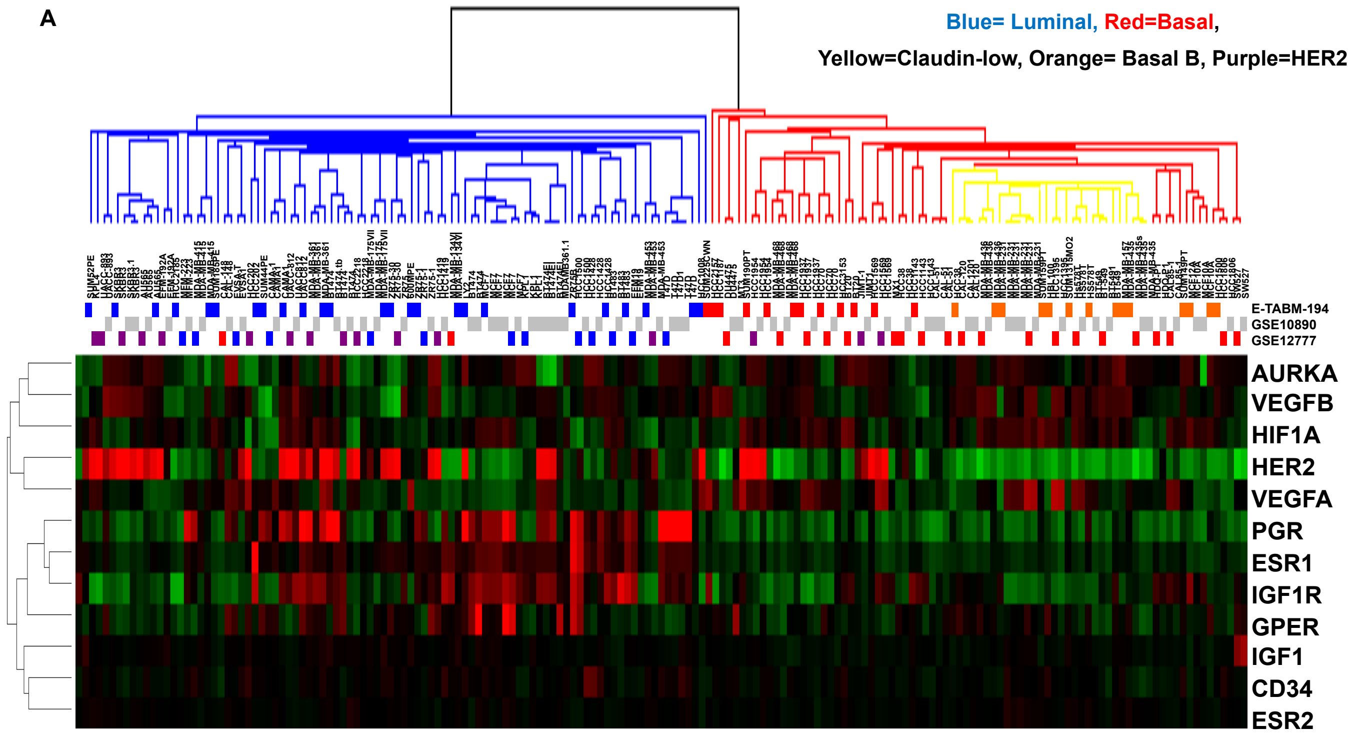

B

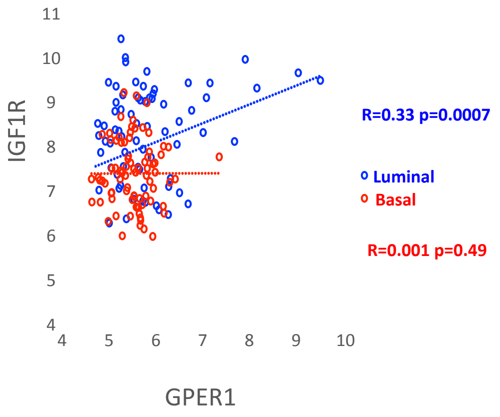

Figure S1
