# Supplementary figures and images for: GPER mediates the angiocrine actions induced by IGF1 through the HIF-1α/VEGF pathway in the breast tumor microenvironment
Source: Breast Cancer Res. 2017 Dec 6;19:129. doi: 10.1186/s13058-017-0923-5 (PMC5719673; doi:10.1186/s13058-017-0923-5)

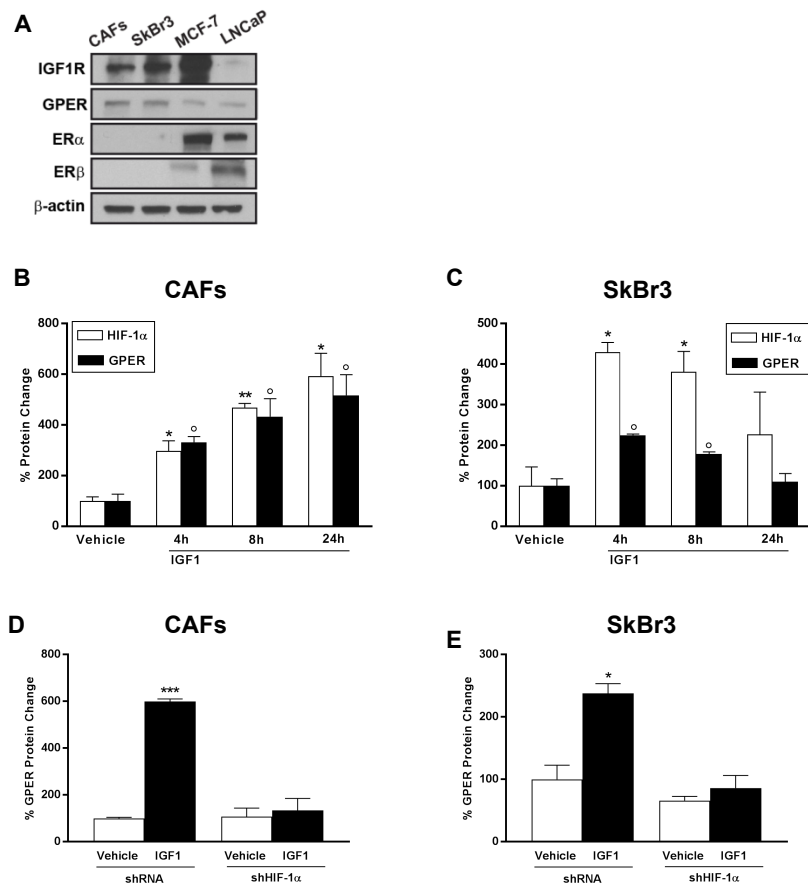

Figure S2

Supplement: Supplementary file 2 — Estrogen receptor expression and densitometric analysis of western blotting. (PDF 1130 kb) [file 13058_2017_923_MOESM2_ESM.pdf]

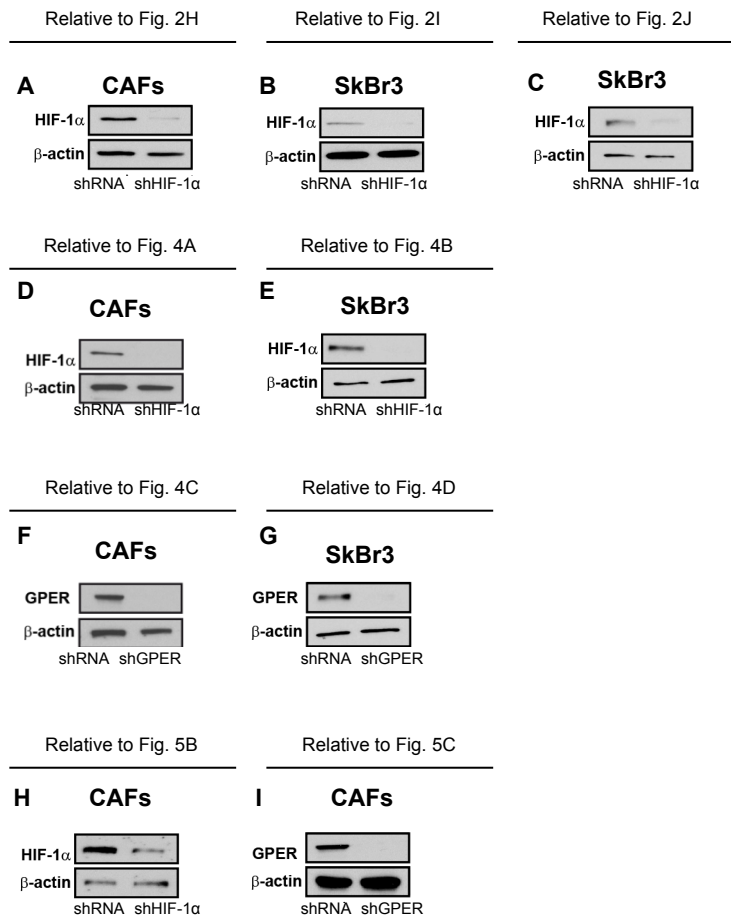

**Figure S3**

Supplement: Supplementary file 3 — Efficacy of HIF-1α and GPER silencing. (PDF 3483 kb) [file 13058_2017_923_MOESM3_ESM.pdf]

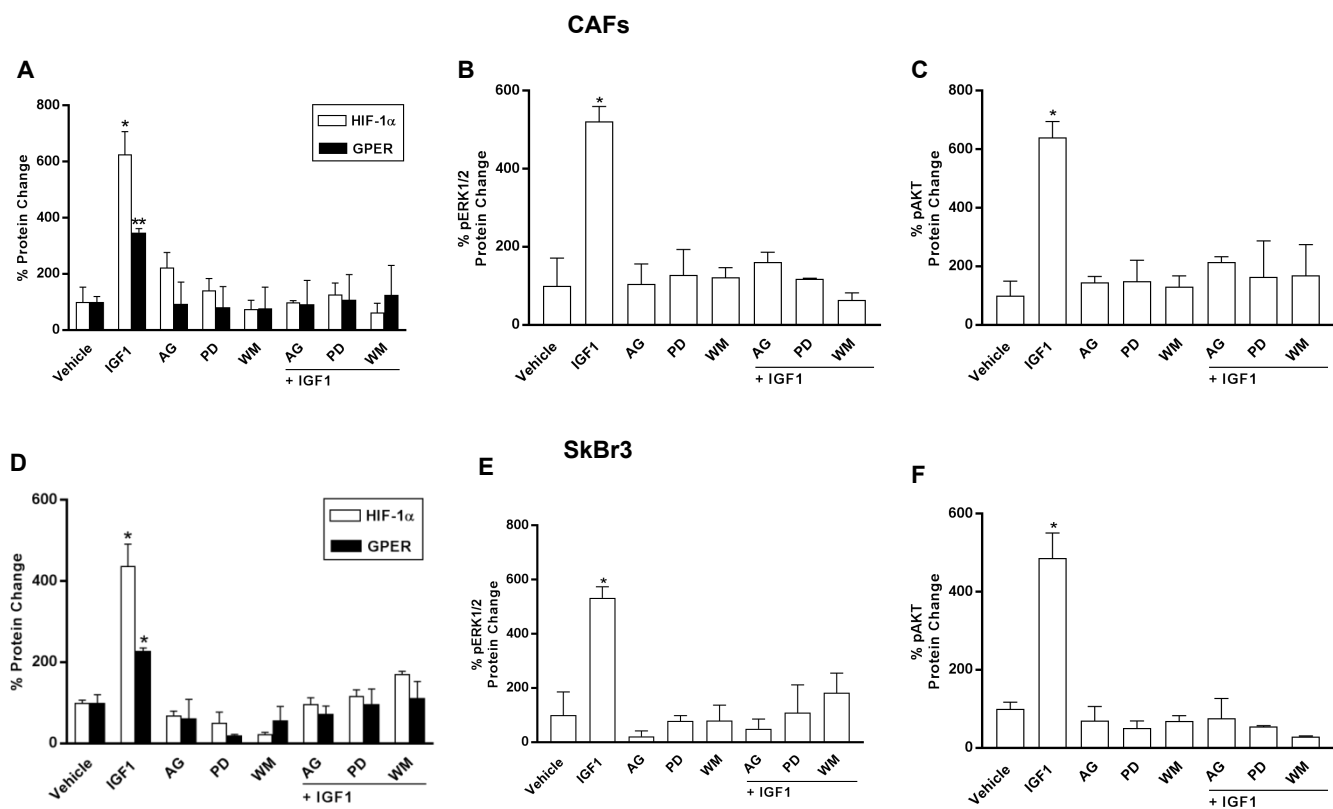

Figure S4

Supplement: Supplementary file 4 — ERK1/2 and AKT activation in SKBR3 cells and CAFs. (PDF 155 kb) [file 13058_2017_923_MOESM4_ESM.pdf]

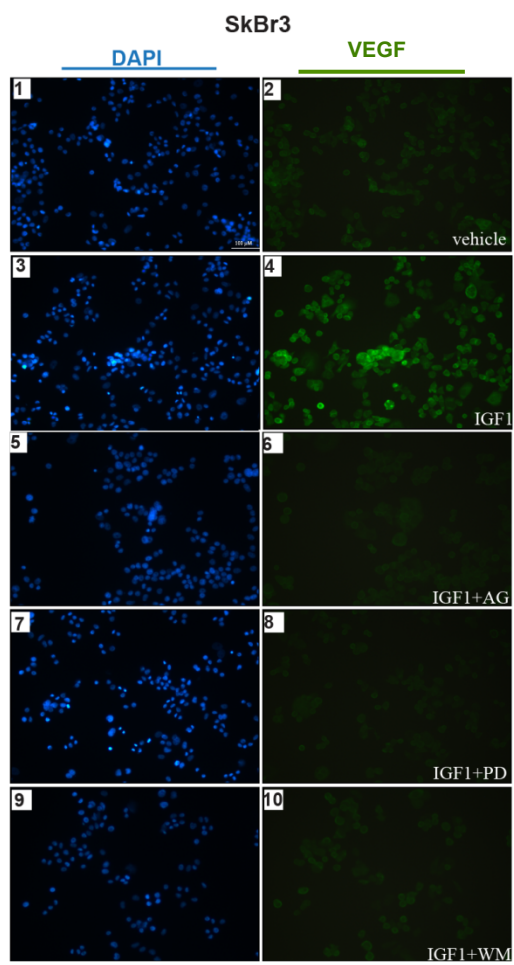

**Figure S5**

Supplement: Supplementary file 5 — IGF1 induces VEGF protein expression in CAFs. (PDF 1335 kb) [file 13058_2017_923_MOESM5_ESM.pdf]

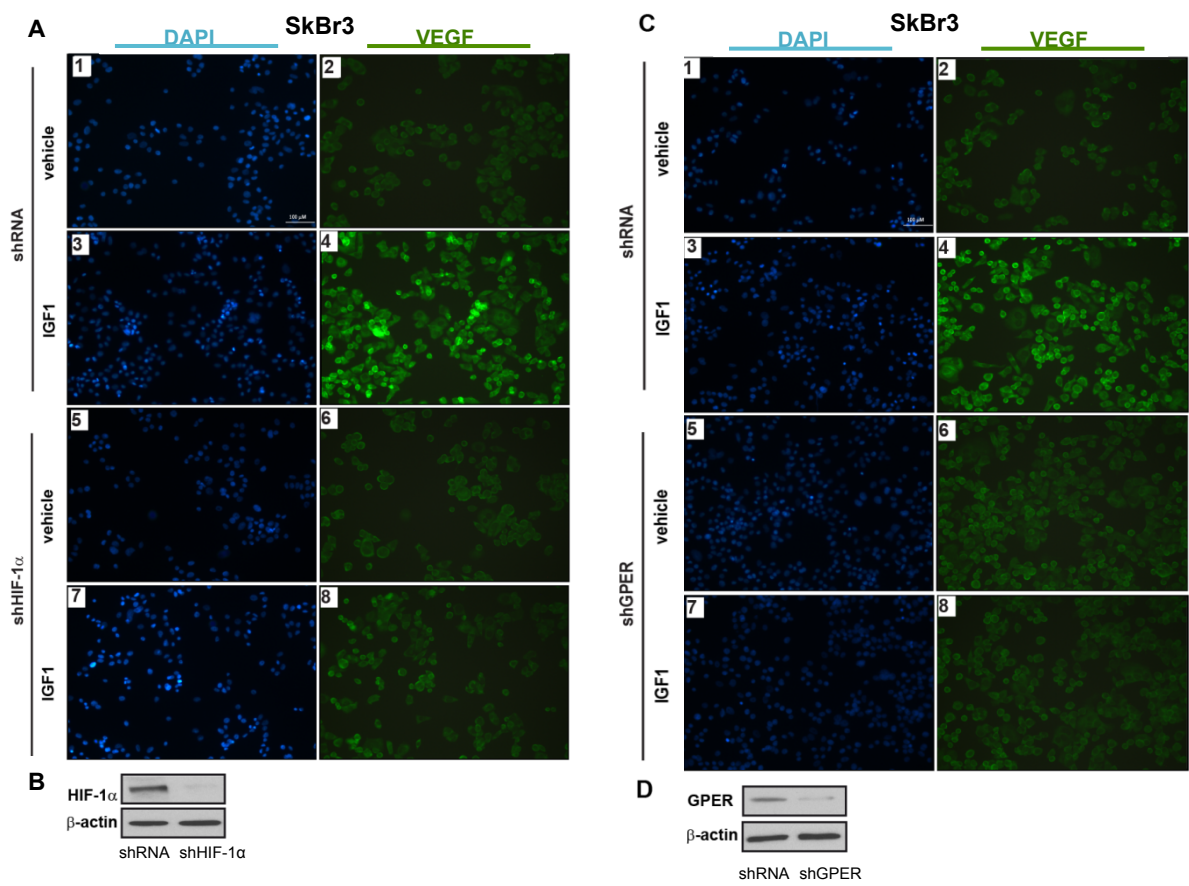

**Figure S6**

Supplement: Supplementary file 6 — HIF-1α and GPER silencing abrogates IGF1-induced VEGF expression in SKBR3 cells. (PDF 2451 kb) [file 13058_2017_923_MOESM6_ESM.pdf]

# Tube formation efficiency in HUVEC

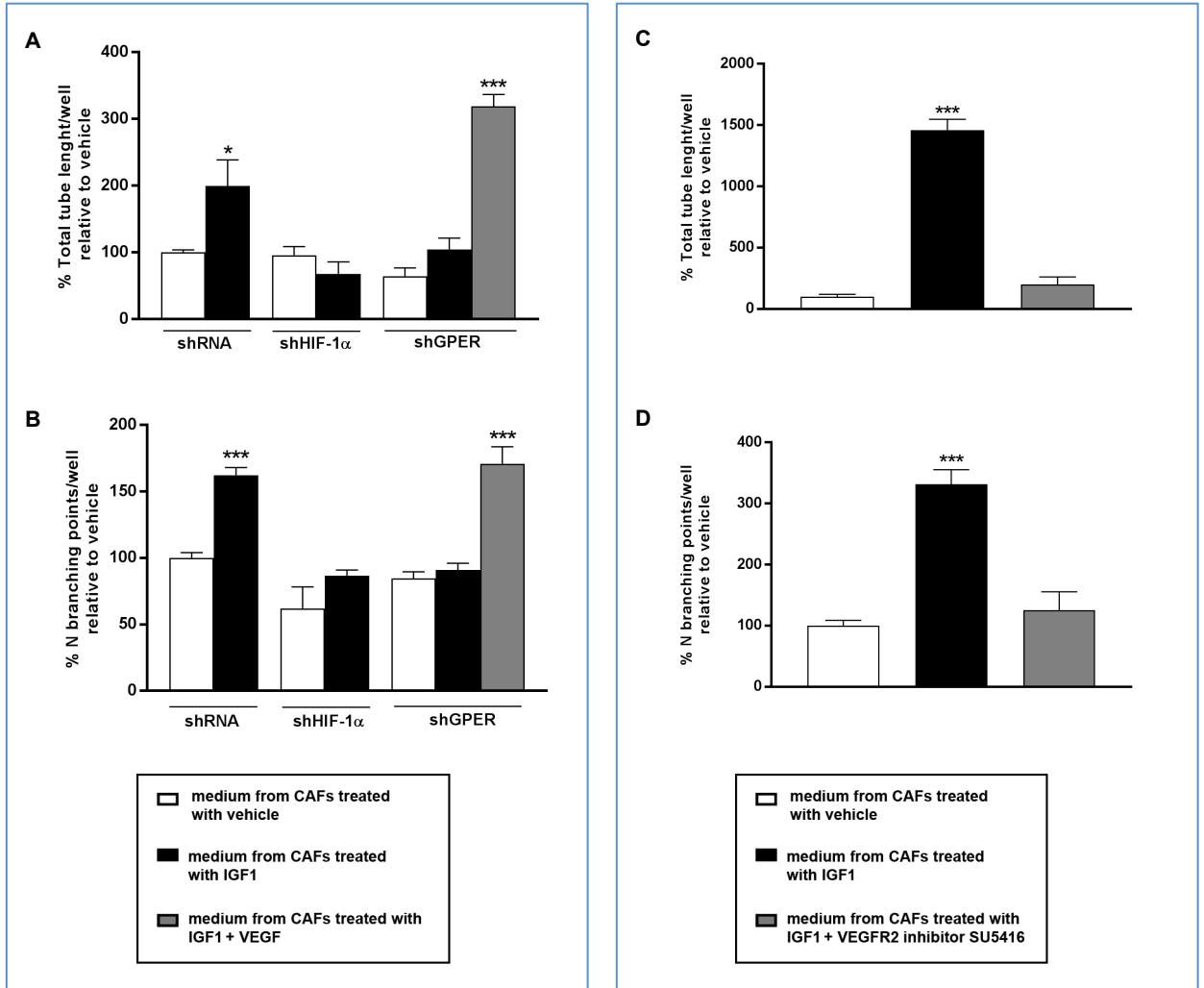

Figure S7

Supplement: Supplementary file 7 — Quantification of tube formation. (PDF 123 kb) [file 13058_2017_923_MOESM7_ESM.pdf]

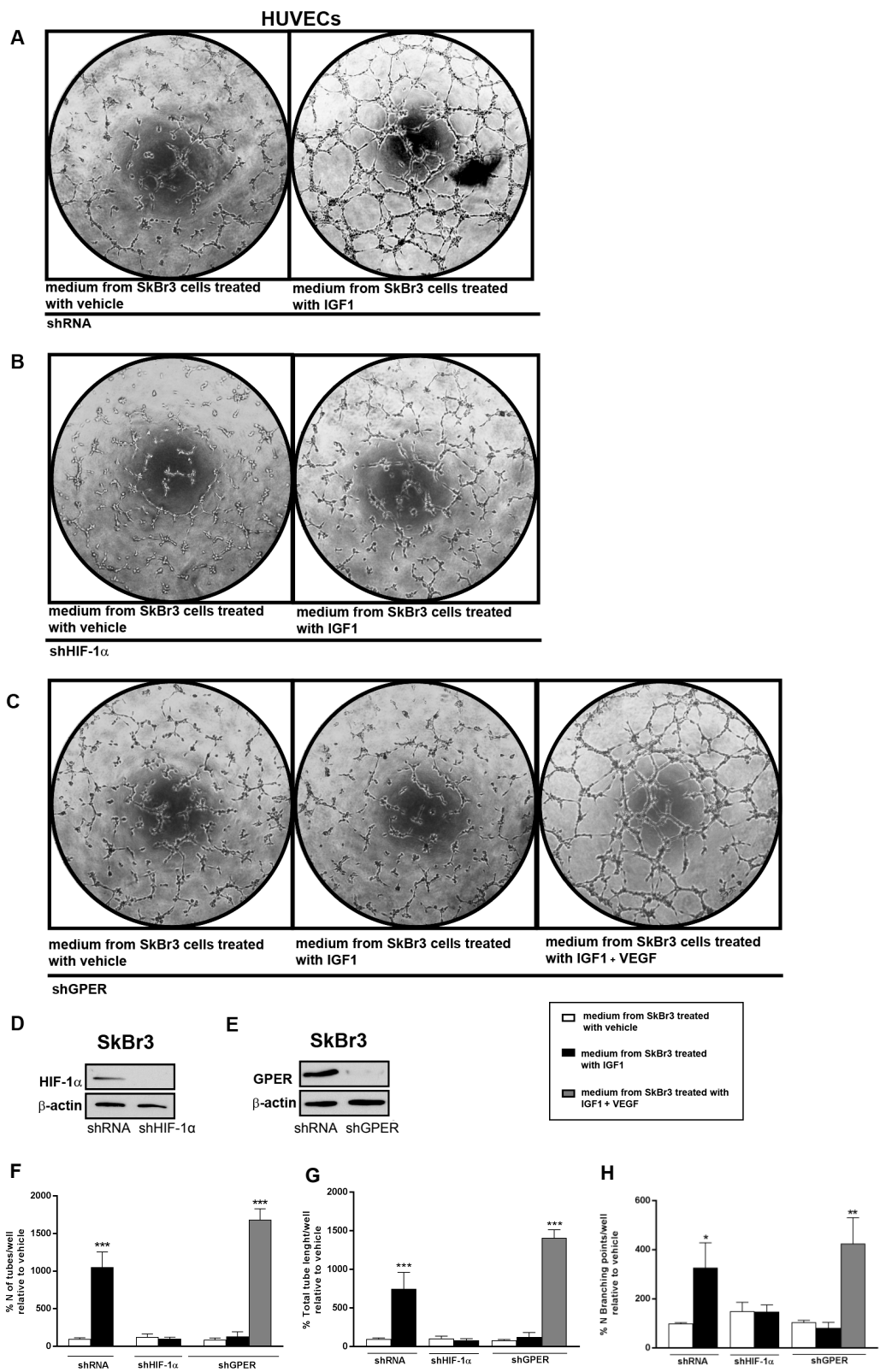

**Figure S8**

Supplement: Supplementary file 8 — IGF1 triggers endothelial tube formation. (PDF 1076 kb) [file 13058_2017_923_MOESM8_ESM.pdf]

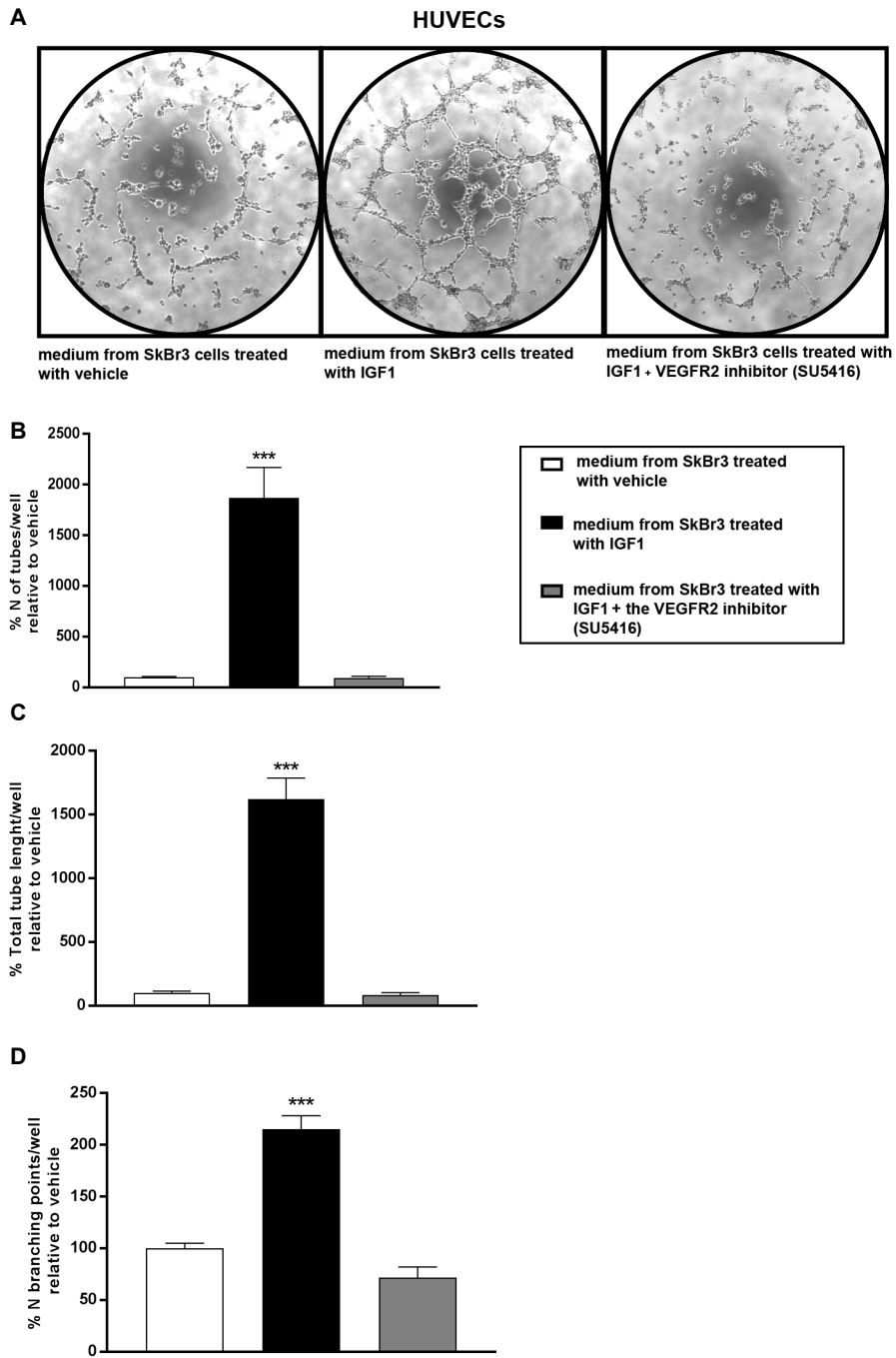

Figure S9

Supplement: Supplementary file 9 — VEGFR2 inhibition prevents endothelial tube formation. (PDF 457 kb) [file 13058_2017_923_MOESM9_ESM.pdf]
